# In silico Methods for Identification of Potential Therapeutic Targets

**DOI:** 10.1007/s12539-021-00491-y

**Published:** 2021-11-26

**Authors:** Xuting Zhang, Fengxu Wu, Nan Yang, Xiaohui Zhan, Jianbo Liao, Shangkang Mai, Zunnan Huang

**Affiliations:** 1 Key Laboratory of Big Data Mining and Precision Drug Design of Guangdong Medical University, Key Laboratory for Research and Development of Natural Drugs of Guangdong Province, School of Pharmacy, Guangdong Medical University, No. 1 Xincheng Road, Songshan Lake District, Dongguan, 523808 China; 2 Hubei Key Laboratory of Wudang Local Chinese Medicine Research, School of Pharmaceutical Sciences, Hubei University of Medicine, Shiyan, 442000 China; 3 The Second School of Clinical Medicine, Guangdong Medical University, Dongguan, 523808 China; 4 Southern Marine Science and Engineering Guangdong Laboratory (Zhanjiang), Zhanjiang, 524023 China

**Keywords:** Therapeutic target, Drug discovery, Target identification, Comparative genomics, Network

## Abstract

At the initial stage of drug discovery, identifying novel targets with maximal efficacy and minimal side effects can improve the success rate and portfolio value of drug discovery projects while simultaneously reducing cycle time and cost. However, harnessing the full potential of big data to narrow the range of plausible targets through existing computational methods remains a key issue in this field. This paper reviews two categories of in silico methods—comparative genomics and network-based methods—for finding potential therapeutic targets among cellular functions based on understanding their related biological processes. In addition to describing the principles, databases, software, and applications, we discuss some recent studies and prospects of the methods. While comparative genomics is mostly applied to infectious diseases, network-based methods can be applied to infectious and non-infectious diseases. Nonetheless, the methods often complement each other in their advantages and disadvantages. The information reported here guides toward improving the application of big data-driven computational methods for therapeutic target discovery.

**Graphical abstract:**

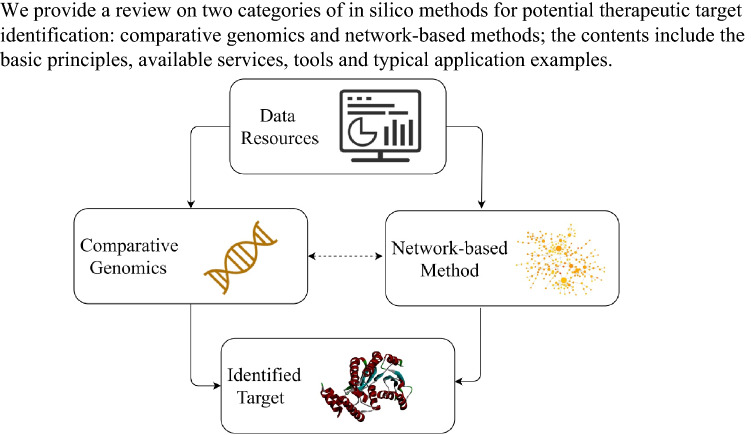

## Introduction

Target identification and validation is the top priority in drug discovery [1]. Molecules or drugs that interact with a rational target or selected combinations of targets have improved odds of therapeutic success. An analysis of AstraZeneca’s drug research and development programs showed that 82% of program terminations in preclinical studies were due to safety issues, of which 25% were target-related [2]. Meanwhile, 48% of safety failures in clinical trials are target-related. Therefore, guidance on the appropriate selection of candidate targets can help improve the success rate and portfolio value of drug discovery projects while also reducing time and cost [3].

Traditionally, target discovery has relied on wet experiments, a process that is time-consuming, expensive, and low in accuracy. With the development of bioinformatics, chemical informatics, and omics, computer-aided therapeutic target discovery methods or in silico methods have come to the fore [4–6]. By integrating big data with computational methods, computer-aided therapeutic target discovery greatly reduces the scope of experimental targets, shortens the drug discovery and development cycle, and reduces the experimental cost. At present, the two main categories of in silico methods for potential therapeutic target identification are comparative genomics [7] and network-based methods [8]. One of many important characteristics differentiating these methods is that comparative genomics is mostly used in infectious diseases, whereas network-based methods can be used not only in infectious diseases, but also in non-infectious diseases. Nonetheless, these categories of methods often complement each other in their advantages and disadvantages.

With the completely sequenced human genome, in addition to the completed genome sequences of many model organisms, there are increasing research-focused efforts to understand the function of a genome and molecular evolution. Finding potential therapeutic targets among cellular functions based on understanding their related biological processes in pathogens and their hosts has become imperative as antimicrobial resistance continues to spread rapidly. To identify therapeutic targets, comparative genomics combines the information contained in genome database resources and software to reveal fatal weaknesses of pathogens that affect their growth and reproduction in the host, such as genes essential for the survival, growth, and important functions of pathogens [9]. In addition, comparative genomics can also filter out homologs by comparing genomes of pathogens and hosts, avoiding the toxic and side-effects of newly designed drugs on the host, in turn, increasing the success rate of drug design [9].

With many pathogenic variants associated with disease in non-coding regions or difficult to target genes, the number of associations that are candidates for development into drugs is limited. Approaches that combine data from pathway databases or biological networks can broaden the number of potential targets to increase the number of associations that lead to effective treatments. As such, network-based strategies are among the state-of-the-art computation models for target identification and are also an important bridge connecting network pharmacology [10], network medicine [11], network biology [12], systems biology [13], and multi-omics data. By combining pathway analysis and the network graph theory concept, network-based strategies not only focus on the interactions (edges) between individual molecules (nodes) and coordinated pathways but also enable a systematic visual exploration of the biological (or biomedical) networks to identify the components of functional importance in the network. In this regard, network-based methods are invaluable in identifying biomarkers, discovering disease diagnosis targets, and finding potential therapeutic targets [14]. The main concept of network-based methods is to map all the relevant data to a visual network. Highly connected nodes (central nodes) that act as bridges between consecutive network components in a single network are predicted as essential proteins or genes of the pathogen (or biological process) and shown to be related to the modular structure of the physical and functional interaction network. Such nodes are hypothesized to be ideal therapeutic targets in the network because they maintain the network integrity [8]. Meanwhile, by searching for highly differential nodes in different networks, those nodes that specifically exist in disease cells can also be hypothesized as potential therapeutic targets [15].

**Fig. 1:**
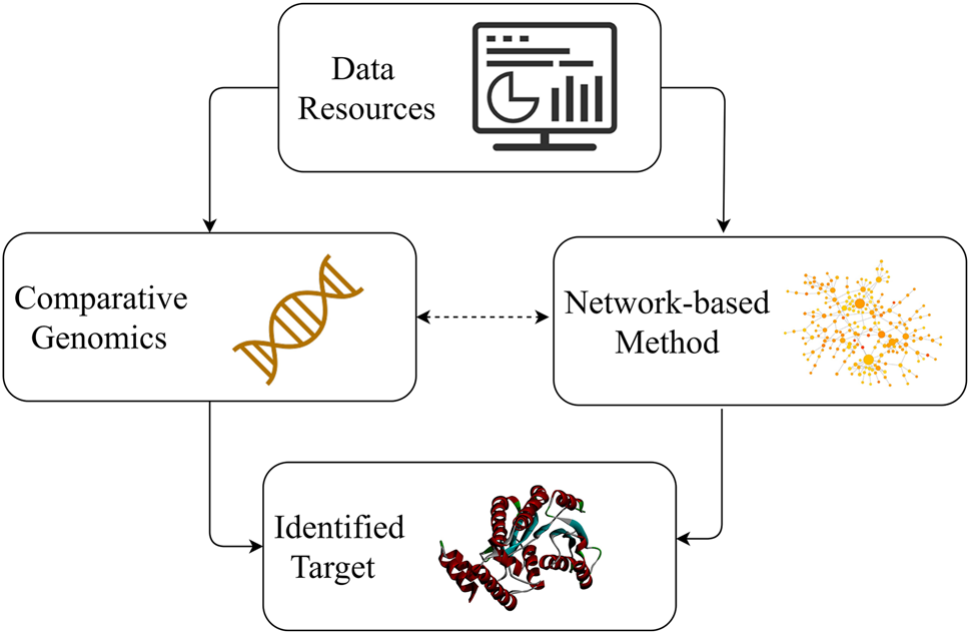
Simplified workflow of in silico methods for identification of potential therapeutic targets

Here, we provide a detailed review of the rationales of comparative genomics and network-based methods for the in silico identification of potential therapeutic targets (Fig. 1). We describe the commonly used databases, software, and applications and discuss these methods in the context of their advantages and disadvantages, contrasts and similarities, comparison with related target identification methods, and relevant published reviews and prospective studies. The information provided in this review will help readers and researchers quickly understand the rationales of in silico therapeutic target identification methods that could further advance research in this area.

## In silico Comparative Genomics and Network-Based Methods

### Comparative Genomics Methods

In the past two decades, whole-cell screening (including large numbers of genetic screening) and in vitro screening of synthetic libraries have been used to identify novel lead compounds with powerful antimicrobial properties [16]. With the completely sequenced human genome, in addition to the completed genome sequences of numerous bacteria and fungi, the number of genes has been rapidly growing. Probing and comparing sequence characteristics between and within species have become a part of most biological queries [17]. Comparative genomics [7] and the recently emerged subtractive genomics (described later in Sect. 6.5) [18] are useful tools for the identification of potential therapeutic targets, such as conserved genes [17] and putative essential genes [9] that affect cell viability in pathogens. Comparative genomics approaches are based on the hypothesis that potential targets are critical in the survival of pathogens and constitute a key component of their metabolic pathways [19]. Moreover, to eliminate deleterious host responses, the target should have no conserved homolog in the human host [20]. Spaltmann et al. proposed two criteria for a gene to be considered a therapeutic target. First, the gene must be necessary for the survival and growth of the pathogen, thereby improving the therapeutic effect of the drug acting on the target. Second, the gene should exist in pathogens but not in mammals; in this way, the drug would have the potential to become a broad-spectrum antimicrobial agent [21]. A gene that meets these criteria can be found using a comparative genomics approach.

**Fig. 2:**
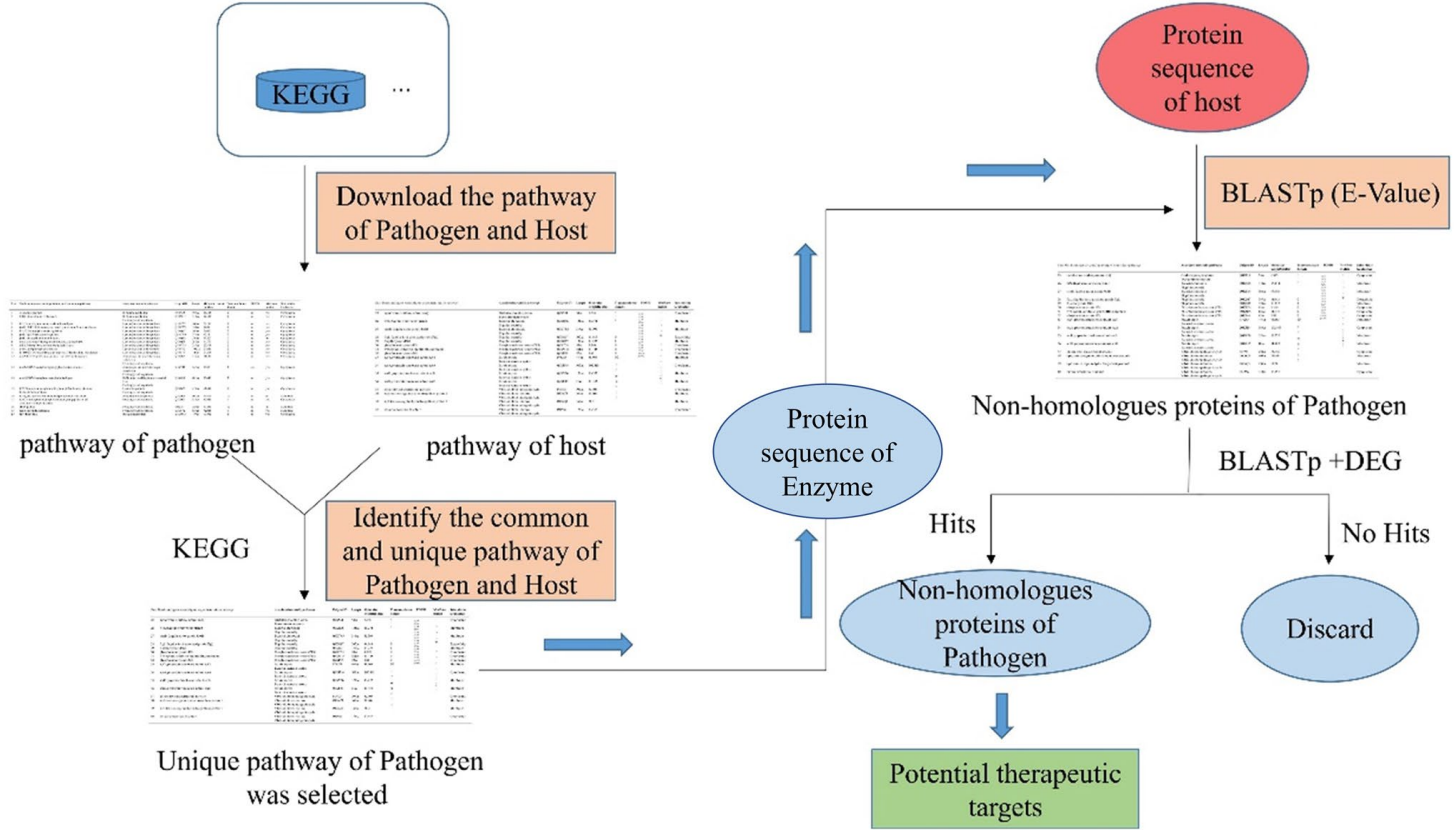
The workflow of identifying potential therapeutic targets by comparative genomics

In Fig. 2, we have summarized the three main steps involved in comparative genomics-based identification of therapeutic targets [22]. The first step is the collection of metabolic pathway enzymes or essential genes of pathogens. It involves obtaining all the metabolic pathways that exist both in the host and pathogen from the Kyoto Encyclopedia of Genes and Genomes (KEGG) Pathway Database [23]. Then, all pathogen pathways are compared with host pathways to determine any overlap [22]. Next, the metabolic pathways are classified. Pathways existing in both the pathogen and the host are removed and named shared pathways, while those existing in the pathogen but not in the host are pooled and named unique pathways [19]. Finally, the gene names and identification of all involved enzymes in the shared and unique pathways are identified and collected from the KEGG Genes Database [22].

Step two is the retrieval analysis of the protein sequences and the use of the basic local alignment search tool (BLAST). First, the protein sequences of all enzymes involved in unique pathways are retrieved from the Universal Protein Resource (UniProt) database [24] in FASTA format. Then, each protein sequence is submitted to a BLASTp analysis (a protein–protein analysis that compares an amino acid sequence against a protein sequence database; discussed in further detail in Sect. 4.1) against the sequences of enzymes in the host metabolic pathways at a set E-value cutoff, the threshold to define a BLAST “hit.” BLAST results with no hits with host enzymes are identified as non-homologous enzymes of the pathogen [9].

The third and final step in the comparative genomics-based identification of therapeutic targets is the identification of essential non-homologous enzymes in the pathogen. To achieve this, the BLASTp analysis is carried out in the database of essential genes (DEG). The protein sequences with significant homology in the DEG database are described as protein sequences vital to the pathogen's survival [18].

Therapeutic targets identified by comparative genomics methods have two essential characteristics. One, the selected targets have significant impacts on some important physiological functions of the pathogen, ensuring the effectiveness of the newly designed drug. Two, by comparing the protein sequences between potential therapeutic targets and the host to identify whether there is homology, any toxic side effects on the human body when the drug interacts with the target can be avoided, in turn, improving the safety of the pharmacological effects of new drugs [20].

### Network-Based Methods

The reason the network-based method can be used for therapeutic target identification is based on the assumption that the influence of specific locations in a biological network can spread along the edges (interactions) of the network [11]. The rationales of network-based methods for predicting therapeutic targets are centrality and differentia. Centrality refers to the analysis of network topological parameters when building a single network. A node in a more central position indicates that it plays a more integral in the network. For example, it may be an essential protein for pathogen survival and thus identified as a potential therapeutic target [8]. However, centrality sometimes cannot be applied directly to normal human protein networks because of the toxicity of acting on such critical nodes [10, 25]. To solve this problem, the direct screening and elimination process of homologous proteins involved in metabolism can be complemented with differential network analysis in which two or more networks are compared, such as normal cell and disease (mostly cancer) cell networks, different subtype networks of cancer, and tissue-specific networks. In this way, the node sets specific to disease cells or highly differential between networks are obtained and identified as potential therapeutic targets [26]. Differential network analysis can also screen out targets that exist in disease cells but not in normal cells or targets connected differentially in different networks to make the identified targets more selective, thereby improving therapeutic security. The highly differential nodes obtained in this way can be further analyzed using network topology to obtain highly centralized nodes that have been double-screened, increasing the reliability of the identified nodes [27].

**Fig. 3:**
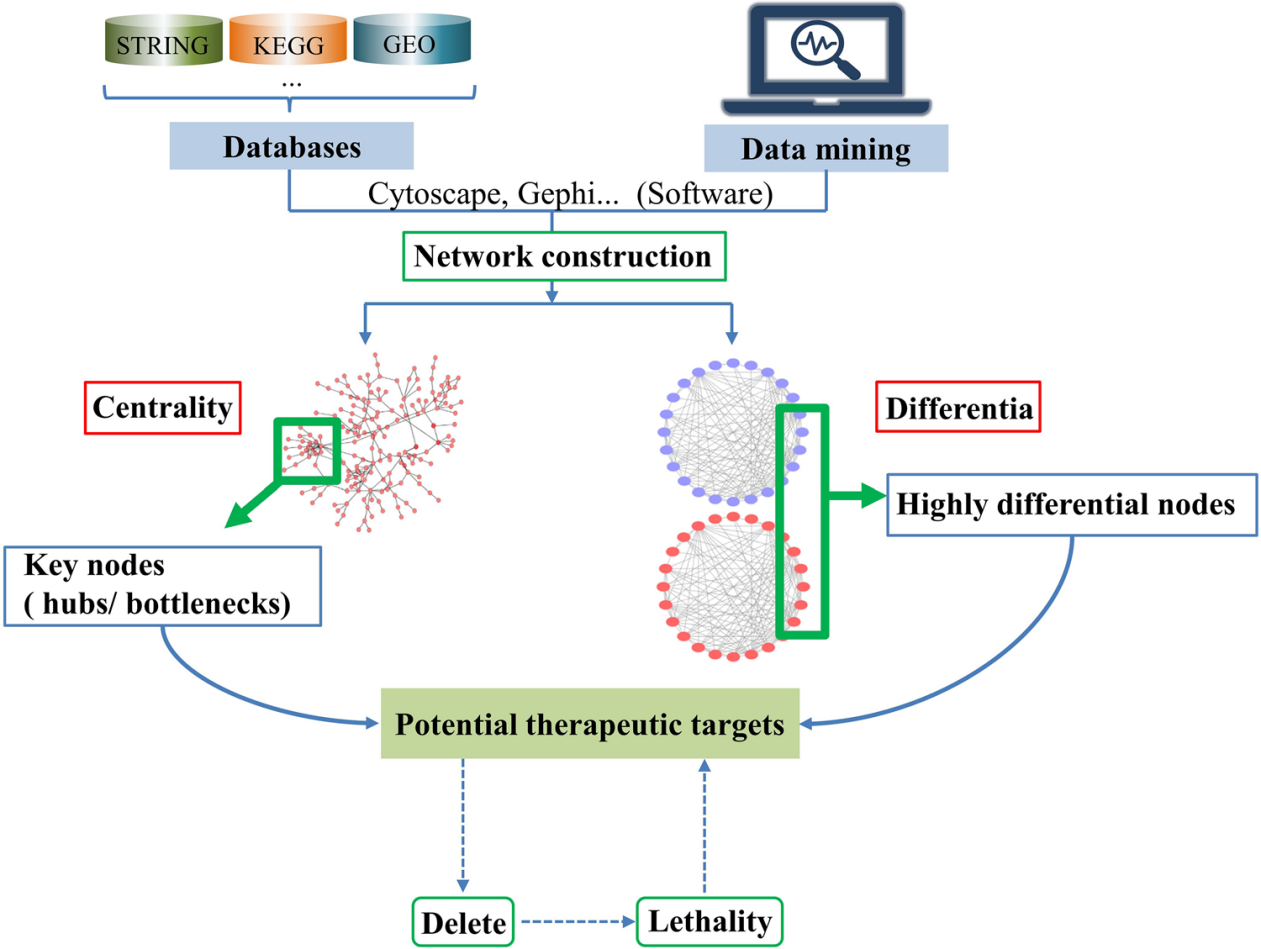
Simplified rationale and approaches of network-based methods for potential therapeutic target identification

According to the rationales of centrality and differentia, network-based methods can be divided into two approaches: the centrality-based approach and the differentia-based approach (Fig. 3). The first step in both approaches is network construction. Network construction refers to obtaining a large number of relevant data sets through data mining [28] or from various databases, websites, and experimental data and carrying out attribute mapping through network visualization tools, namely comprehensive data visualization [29]. Some types of constructed networks are protein–protein interaction (PPI) networks [30], gene interaction networks [31], and miRNA–mRNA interaction networks [32]. After the network is built, the processes of the two approaches diverge.

The centrality-based approach uses some network analysis tools to (i) analyze the topological parameters of nodes in networks and (ii) select nodes with high degree centrality (hub nodes) and high betweenness centrality (bottlenecks), which are often integral in networks and thus can be selected as potential therapeutic targets [33]. The degree centrality of a node refers to the number of direct connections the node has with other nodes in the network [11], while the betweenness centrality of a node refers to the number of shortest paths that pass through the node in the network [34]. The centrality-based approach is most suitable for rapidly growing cells, such as pathogens and cancer cells [8]. In addition to the widely-used degree centrality and betweenness centrality, other parameters, such as closeness centrality, clustering coefficient, average shortest path, eigenvector centricity, and spectral gap centricity, can also be used as centrality indices to predict the importance of nodes, and thus to identify potential therapeutic targets [35, 36]. For further understanding of the definitions of the parameters mentioned above, two references are recommended [35, 36].

As mentioned above, differential network analysis requires the construction of two or more networks, including normal and disease cell networks [30] or networks of different subtypes of cancer [37]. After the construction of networks is completed, some algorithms can be applied to identify differential components between networks, to select nodes that exist in disease cell networks but not in normal cell networks, or to select nodes that are highly differentially connected between or among networks, as predicted potential therapeutic targets [26, 38].

Potential targets identified through centrality and differentia can be further prioritized by observing the lethality of the network when those nodes are removed [39]. Generally, network lethality after removal of a node is positively correlated with the connectivity of the node. When nodes with high degree centrality are deleted, the network diameter will increase rapidly [40]. When nodes with high betweenness centrality are deleted, (i) the average path length will decrease rapidly [41]; (ii) network topology, such as the characteristic path length, will change significantly; (iii) the ability of the remaining nodes to communicate with each other will be weakened, and (iv) the network will disintegrate [42]. Therefore, the more lethal the removal of a node to the network, the more important the node's role, and the greater its potential as a therapeutic target [39].

## Databases

Data acquisition is indispensable to any research work. Therefore, we summarized the databases useful in comparative genomics and network-based methods for identifying potential therapeutic targets. Although some databases can be used for both types of in silico methods, we placed them in separate tables because the most popular features of these databases differ between the two approaches.

### Comparative Genomics

The relevant databases for comparative genomics can be roughly divided into two categories: (i) general databases; those usually used in comparative genomics, such as DEG, KEGG [23], and UniProt; and (ii) specific databases, which mainly provide pathogenic gene sequences of bacteria and fungi, such as the Tuberculosis Database (TBDB), WormBase, and the Virulence Factors of Pathogenic Bacteria Database (VFDB). Table 1 lists the general and specific databases with brief descriptions, including the coverage, availability, latest update, and URL.

**Table 1:** General and specific databases for comparative genomics

Database	Description	Coverage	Availability	Latest update	URL	References
UniProt^a^	A comprehensive resource for protein sequence and annotation data	305,529 proteomes	Free	2020	https://www.uniprot.org/	[24]
UniProtKB/Swiss-Prot^a^	A high-quality annotated and non-redundant protein sequence database	564,277 proteins	Free	2021	https://www.uniprot.org/uniprot/?query=reviewed:yes	[43]
DEG^a^	A database hosting records of currently available essential genomic elements	53,885 essential genes and 786 essential non-coding sequences	Free	2017	http://tubic.tju.edu.cn/deg/	[44]
pDEG^a^	A database contains many details of the predicted essential genes of 16 *Mycoplasma* genomes	5,880 essential genes	Free	2011	https://origin.tubic.org/pdeg	[45]
OGEE^a^	An essentiality database that includes essential and non-essential genes from large-scale experiments	127 gene essentiality experiments for 91 species, 38,822 genes covered by multiple experiments	Free	2021	http://ogeedb.embl.de	[46]
KEGG^a^	A database resource that integrates genomic, chemical and systemic functional information	Four categories (systems, genomic, chemical, and health information) from 18 databases	Free	2021	https://www.kegg.jp/	[47]
PMDB^a^	A public resource aimed at storing manually built three-dimensional models of proteins	Contains > 74,000 models for approximately 240 proteins	Free	2011	http://www.caspur.it/PMDB	[48]
ModBase^a^	A database of comparative protein structure models	Almost 30 million reliable models for domains in 4.7 million unique protein sequences	Free	2013	https://modbase.compbio.ucsf.edu/modbase-cgi/index.cgi	[49]
RefSeq^a^	A comprehensive, integrated, non-redundant, well-annotated database of sequences, including genomic DNA, transcripts, and proteins	197,232,209 proteins, 36,514,168 transcripts, and 108,257 organisms	Free	2021	https://www.ncbi.nlm.nih.gov/refseq/	[50]
Tuberculosis Database^b^	An online database provides integrated access through a single portal to sequence data and annotation, expression data, and literature curation for tuberculosis	Genome sequences of 20 strains	Free	2021	https://www.tbdb.org/	[51]
WormBase^b^	A database about *Caenorhabditis elegans* genome	*C. elegans* and other *Caenorhabditis* genomes	Free	2017	https://wormbase.org//#012-34-5	[52]
VFDB^b^	A database of virulence factors of various medically significant bacterial pathogens	Virulence factors of bacterial pathogens	Free	2021	http://www.mgc.ac.cn/VFs/main.htm	[53]

^a^Denotes general database

^b^Denotes specific database

DEG is a commonly used database in comparative genomics that contains 53,885 essential genes and 786 non-coding essential sequences critical to the survival and growth of bacteria, archaea, and eukaryotes for homology analyses [44]. DEG 15 is the most recent version of this database. It is worth noting that DEG has multiple built-in tools for data analysis and display, such as a subcellular location and distribution analysis tool, a pathway and genomics enrichment analysis tool, and a Venn maps generation tool for comparing genomes between experiments [54].

TBDB is an online platform for basic scientific research on tuberculosis and drug and vaccine discovery and development research. It contains genome sequence data and microarray and RT-PCR expression data, including over 3,000 *Mycobacterium tuberculosis* (*Mtb*) microarrays (2,700 from humans and mice and 260 for *Streptomyces coelicolor*) and 95 RT-PCR datasets, for numerous strains of *Mtb*, as well as data for more than 20 *Mtb*-related strains from in vitro tuberculosis-related experiments and tuberculosis-infected tissues. A wide range of tools is incorporated in the database for browsing, analyzing, searching, and downloading the data [51].

### Network-Based Methods

There are many databases used in network-based methods. We roughly divided the databases into two categories: direct databases and indirect databases. Direct databases cover the interaction data and can be directly imported into network visualization software for network construction. Examples are the Search Tool for Retrieval of Interacting Genes/Proteins (STRING) [55] and the Molecular INTeraction (MINT) database [56]. Indirect databases do not directly cover interaction data but provide detailed annotation of network nodes allowing an in-depth exploration of the network. Some examples include the gene expression omnibus (GEO) [57] and DrugBank [58]. Table 2 (direct databases) and Table 3 (indirect databases) list the databases commonly used in network-based methods, with brief descriptions, including the coverage, availability, latest update, and URL.

**Table 2:** Frequently used direct databases in network-based methods

Database	Description	Coverage	Availability	Latest update	URL	References
STRING	A database of known and predicted protein–protein interactions	24,584,628 proteins from 5090 organisms	Free	2019	http://string-db.org	[55]
MINT	A database designed to store data on functional interactions between proteins	647 organisms and 131,695 interactions	Free	2012	https://mint.bio.uniroma2.it/	[56]
HPRD	A centralized platform to visually depict and integrate information pertaining to domain architecture, post-translational modifications, interaction networks, and disease association for each protein in the human proteome	41,327 protein–protein interactions	Free	2010	http://hprd.org/	[59]
IntAct	Provides an open-source database system and analysis tools for molecular interaction data	119,281 interactors and 1,130,596 interactions	Free	2021	https://www.ebi.ac.uk/intact/	[60]
BioGRID	A comprehensive biomedical resource of curated protein, genetic, and chemical interactions	2,005,220 protein and genetic interactions, 29,093 chemical interactions	Free	2021	https://thebiogrid.org/	[61]
DIP	Catalogs experimentally determined interactions between proteins	28,850 proteins and 81,923 interactions	Free	2020	https://dip.doe-mbi.ucla.edu/dip/Main.cgi	[62]
STITCH	A database of known and predicted interactions between chemicals and proteins	9,643,763 proteins from 2,031 organisms	Free	2016	http://stitch.embl.de/	[63]
miRTarBase	A database of comprehensively annotated, experimentally validated miRNA–target interactions	422,517 curated miRNA–target interactions from 4076 miRNAs and 23,054 target genes	Free	2018	http://miRTarBase.cuhk.edu.cn/	[64]
TarBase	A database of experimentally supported miRNA–gene interactions with detailed information for each interaction	56 tissues, 516 cell types, and 665,843 interactions	Free	2017	http://www.microrna.gr/tarbase	[65]

**Table 3:** Frequently-used indirect databases in network-based methods

Database	Description	Coverage	Availability	Latest update	URL	References
GEO	A public functional genomics data repository supporting microarray experiment (MIAME)-compliant data submissions	162,671 series and 4,777,869 samples	Free	2021	https://www.ncbi.nlm.nih.gov/geo/	[57]
DrugBank	A comprehensive database containing information on drugs and drug targets	14,315 drugs, 4885 targets, and 18,866 drug–target associations	Free	2021	https://go.drugbank.com/	[58]
KEGG	A database resource that integrates genomic, chemical, and systemic functional information	Four categories (systems, genomic, chemical, and health information) from 18 databases	Free	2021	https://www.kegg.jp/	[47]
UniProt	A comprehensive resource for protein sequence and annotation data	305,529 proteomes	Free	2021	https://www.uniprot.org/	[24]
GO	A database source of information on the functions of genes	7,934,369 annotations	Free	2021	http://geneontology.org/	[66]
DAVID	Provides a comprehensive set of functional annotation tools for investigators to understand the biological meaning behind a large list of genes	Information on > 1.5 million genes from > 65,000 species	Free	2020	https://david.ncifcrf.gov	[67]
OMIM	A comprehensive, authoritative compendium of human genes and genetic phenotypes	6,799 phenotypes for which the molecular basis is known and 4,370 genes with phenotype-causing mutation	Free	2021	https://omim.org/	[68]
CARD	A bioinformatic database of resistance genes, their products, and associated phenotypes	88 pathogens and 222,011 alleles	Free	2021	https://card.mcmaster.ca/	[69]
DO	A standardized ontology for human diseases providing descriptions of human disease terms, phenotype characteristics, and related medical vocabulary disease concepts	> 10,500 disease terms and > 7,500 disease terms defined	Free	2021	http://www.disease-ontology.org/	[70]
TTD	A database to provide information about the known and explored therapeutic protein and nucleic acid targets, the targeted disease, pathway information, and the corresponding drugs directed at each of these targets	37,316 drugs and 3,419 targets	Free	2020	http://db.idrblab.net/ttd/	[71]
GeneCards	Provides comprehensive, user-friendly information on all annotated and predicted human genes	42,087 HUGO Gene Nomenclature Committee (HGNC)-approved, 20,916 protein-coding, 219,587 RNA genes	Free	2020	https://www.genecards.org/	[72]
DEG	A database hosting records of currently available essential genomic elements	53,885 essential genes and 786 essential non-coding sequences	Free	2017	http://tubic.tju.edu.cn/deg/	[44]
NDARO	A database of antimicrobial resistance data	> 300,000 pathogen isolates	Free	2020	https://www.ncbi.nlm.nih.gov/pathogens/antimicrobial-resistance/	\
PATRIC	Provides integrated data and analysis tools to support biomedical research on bacterial infectious diseases	Genome metadata from > 60 fields, three types of protein families	Free	2020	https://www.patricbrc.org/	[31]

STRING [55] is the most commonly used direct database in network-based methods. It houses a large number of known and predicted PPIs, including both physical and functional interactions. The data come from the following five main sources: genomic context analysis, high-throughput experimental data, conserved co-expression, artificial text mining, and known information in databases [55]. At the time of writing, STRING covers 24,584,628 proteins from 5090 organisms [55]. This database provides an intuitive and fast viewer for online use, supports online network visualization, and provides a user-friendly platform for data integration with knowledge from other public resources [55].

The GEO database [57] is the most commonly used indirect database in network-based methods. It is a universal public repository for archiving and freely distributing high-throughput microarray, next-generation sequencing, and other forms of high-throughput functional genomic data, with complete and clear annotations from the research community [57]. To date, the GEO database covers 162,671 series comprising 4,777,869 samples. It provides a powerful search engine for users to identify, analyze, and visualize related data of interest. It also supports sophisticated field queries, sample comparison applications, and gene expression profiles [57].

## Software and Tools

### Comparative Genomics

Table 4 lists the software and tools used in comparative genomics to identify targets. Brief descriptions, availability, the latest update, and the URL are also provided. In comparative genomics, the BLAST suite (BLASTn, BLASTp, BLASTx, tBLASTn, and tBLASTx) is widely used to analyze the functional and evolutionary relationship between nucleic acid and protein sequences [73]. BLAST is a free online tool that can also be downloaded offline from the National Center for Biotechnology Information (NCBI) website. BLASTn is for nucleic acid sequence alignment; BLASTp is for protein sequence alignment; BLASTx compares the six-frame conceptual translation products of a nucleotide query against a protein sequence database; tBLASTn compares a protein query sequence against a sequence database dynamically translated in all six reading frames, and tBLASTx compares the six-frame translation of a nucleotide query sequence against the six-frame translations of a nucleotide sequence database [73, 74]. There are many specific search modules in NCBI besides those regular modules. For example, smartBLAST [75] can be used to query highly similar proteins, GlobalAlign module to compare two sequences in the entire sequence, CD-search [76] to find conservative domains in a sequence, and CDART to query sequences with similar conservative domain architecture [77]. Moreover, NCBI provides an independent program BLAST + for users that dramatically accelerates the speed of long sequences query and chromosome length databases query to address the problem of slow-speed BLAST online comparison [78]. Recently, Du et al. designed a cross-platform local BLAST visualization software developed in Python using the in-built graphical user interface (GUI) module TKinter [79]. BlastGUI, as it is known, utilizes BLAST + as a comparison tool to perform the local operation and sequence comparison visualization. This user-friendly tool allows users without familiarity in computational coding and basic computer skills to compare a sequence directly without additional formatting efforts [79]. BlastGUI preprocesses the input sequence, so the computational complexity of sequence comparison is low. To carry out the comparison, the user enters the file in FASTA format into the search box of BLAST. The maximum acceptable length of nucleotide and protein sequences is generally 1000–2000, and the maximum molecular weight of the protein is 10 to 100 kD. The sequence information can be obtained from NCBI free of charge. Alternatively, the NCBI BLAST uses the indirect BLAST algorithm to run a large number of BLAST searches without using a browser, and the comparison results are returned by e-mail [73].

**Table 4:** Software and tools of comparative genomics in the identification of potential therapeutic targets

Software and tools	Description	Availability	Latest update	URL	References
ClustalW/ ClustalX	Multiple alignment of nucleic acid and protein sequences	Free	2010	http://www.clustal.org/clustal2/	[80]
Clustal Omega	Fast, scalable generation of high-quality protein multiple sequence alignments using Clustal Omega	Free	2016	http://www.clustal.org/omega/	[81]
MUSCLE	One of the best-performing multiple alignment programs according to published benchmark tests, with accuracy and speed that are consistently better than ClustalW	Free	2020	http://www.drive5.com/muscle/	[82]
Jalview	A program for multiple sequence alignment editing, visualization, and analysis	Free	2020	http://www.jalview.org/	[83]
KAAS	A web-based server automatically assigns *K-*numbers to genes in the genome, enabling reconstruction of KEGG pathways and BRITE hierarchies	Free	2015	http://www.genome.jp/kegg/kaas/	[84]
CD-HIT	A program for clustering and comparing protein or nucleotide sequences	Free	2015	http://weizhongli-lab.org/cd-hit/	[85]
PGAT	A prokaryotic-genome analysis tool focused particularly on comparing different strains of the same species	Free	2011	http://nwrce.org/pgat	[86]
ESSENTIALS	Software for predicting essential genes by utilizing transposon insertion sequencing analysis	Free	2012	https://trac.nbic.nl/essentials/	[87]

### Network-Based Methods

Table 5 provides brief descriptions, availability, latest update, and URL of software and tools for network-based methods used in previous target identification studies over the past 5 years. Among them, Cytoscape is the most widely used and representative software. Therefore, we chose it as an example for further description of the network-based methods. Cytoscape is a general-purpose platform to analyze and visualize complicated molecular interaction networks. It can be used for integrating massive molecular interaction data. Dynamic states and molecular interactions are mapped as attributes on nodes and edges, and static hierarchical data (such as protein function ontology) are supported by annotations [88]. The Cytoscape Core is the code that organizes, displays, reads, and writes networks but contains no biology-related functionality. It is equipped with basic functionality to lay out and query the network, visually integrate the network with expression profiles, phenotypes, and other molecular states, and link the network to databases of functional annotations [88]. This core functionality is extended by Cytoscape apps. Cytoscape allows users to import attributes from tables whose simplest format are tab-delimited text files containing one column of primary identifiers of network nodes and auxiliary columns of attributes needed mapping to the nodes [89]. To reduce the complexity of a large interaction network, users can create filters based on the attributes as needed and use the Cytoscape built-in function to search [89]. In addition to directly filtering nodes using the built-in topological parameters in Cytoscape, users can also use apps (formerly called plugins), such as stringApp [90], the Biological Networks Gene Oncology (BiNGO) tool [91], Molecular Complex Detection (MCODE) [92], and cytoHubba, a user-friendly interface to explore key nodes and subnetworks [93]. StringApp combines the resources of the STRING database and Cytoscape in the same workflow and facilitates the import of STRING molecular networks into Cytoscape for executing STRING analysis in the script file [90]. BiNGO provides a comprehensive set of annotation tools for Gene Ontology (GO)-level annotations of a variety of organisms. It enables the extraction of information about overexpression of a gene in biological networks and supports user-defined annotations and ontologies [91]. MCODE enables searches for densely connected regions within large PPI networks that may reflect molecular complexes. The method is based on connectivity data [92]. CytoHubba provides a one-stop calculation of 11 topological analysis methods to help users explore hub objects from complex biological networks [93]. These useful apps are freely available from the Cytoscape App Store (http://apps.cytoscape.org/).

**Table 5:** Software and tools of network-based methods for identification of potential therapeutic targets

Software and tools	Description	Availability	Latest update	URL	References
Cytoscape	An open-source platform for complex network visualization and analysis	Free	2020	https://cytoscape.org/	[88]
Gephi	Open-source software for network visualization and analysis	Free	2017	https://gephi.org/	[94]
NetworkAnalyst	A comprehensive network visual analytics platform for gene expression analysis	Free	2021	https://www.networkanalyst.ca/	[95]
HIPPIE	A web tool to generate reliable and meaningful human protein–protein interaction networks	Free	2019	http://cbdm-01.zdv.uni-mainz.de/~mschaefer/hippie/	[96]
PathwayLinker	Assembles validated physical and genetic interaction data with pathway information	Free	/	http://PathwayLinker.org	[97]
KOBAS	A webserver for gene/protein functional annotation and gene set enrichment	Free	2020	http://kobas.cbi.pku.edu.cn/kobas3	[98]
BioCyc	A webserver containing a collection of 18,030 Pathway/Genome Databases (PGDBs), plus software tools for exploring them	Free	2021	https://biocyc.org/	[99]
Cfinder	Software for finding and visualizing overlapping dense groups of nodes in networks	Free	2014	http://cfinder.org/	[100]
Pajek	A program package for the analysis and visualization of large networks	Free	2021	http://mrvar.fdv.uni-lj.si/pajek/	[101]
NetworkX	A Python package for the creation, manipulation, and study of the structure, dynamics, and functions of complex networks	Free	2021	https://networkx.org/	\

## Applications

### Comparative Genomics

With the arrival of the post-genome era, target-based drug design strategy has gradually become the focus [102]. Both the improvement of the sequencing technology and the exponential explosion of the number of fully sequenced genomes has made it possible to select reasonable new therapeutic targets and vaccine candidates throughout the genome. Drug resistance is becoming increasingly widespread due to the continuous evolution of bacterial strains, such as *Streptococcus pneumoniae* and *Mtb*. Knowledge of therapeutic targets and drug candidates is useful for enhanced drug discovery and is becoming increasingly reliant on comparative genomics technology [103]. Table 6 lists recent applications of comparative genomics in finding therapeutic targets. We selected some specific examples to describe in this section.

**Table 6:** Examples of prediction of potential therapeutic targets by comparative genomics in recent years

Databases	Software and tools	Comparative types	Related pathogens/diseases	Predicted targets	References
UniProt, DEG, Swiss-Prot, TIGR	BLASTx	Genes	*Helicobacter pylori*	*H. pylori* essential genes	[20]
PDTD, DSSP	ClustalW, DOCK4.0, TarFisDock	Proteins	*H. pylori*	PDF	[109]
WormBase	BLASTp	Genes	Human fungal	589 essential genes	[110]
NCBI Proteins	ConSurf server, MUSCLE, Jalview	Proteins	Influenza A virus	NS1 protein, NS2 protein	[111]
*Pseudomonas*, PDB	BLAST, ExPASy server, ClustalW, ESPript	Genes	*Pseudomonas*	*DAHPS* sequence	[48]
COGS, DEG, Pathema-JCVI, STRING	BLASTx, BLASTp	Proteins	*Clostridium botulinum*	39% essential proteins	[112]
KEGG, DEG, Swiss-Prot	BLASTp	Proteins	*Actinobacillus pleuropneumoniae*	rpoA, metG, gltX	[113]
NCBI Genome, DrugBank, DEG	BLAST	Proteins	Nontuberculous mycobacteria	15 candidate proteins	[114]

Determining essential genes of pathogens is a common method to identify potential therapeutic targets. For example, Tilahun et al. [104] retrieved the protein-coding genes of *Mtb* from the *Mtb* database and identified the essential genes by a BLAST search of the retrieved protein-coding genes against DEG. Then, the corresponding protein sequences, obtained by searching in DEG, were used to perform a BLASTp search of human protein sequences to avoid host toxicity in the subsequent drug development. Finally, 572 essential genes with no homology to human genes were selected from 3958 genes of *Mtb.* Discovering potential therapeutic targets from the proteins encoded by essential genes can refine the search scope of therapeutic targets. The existence of homologous genes is a powerful predictor of biological importance [105] and a breakthrough in therapeutic target identification. For example, Satya et al. [48] sequenced the gene encoding 3-deoxy-D-arabinoheptulosonate-7-phosphate synthase (*DAHPS*) in *Pseudomonas fragilis* (*Pf*). Sequence analysis showed high homology (84%) of *Pf*-*DAHPS* with other *Pseudomonas DAHPS*, indicating that it was possible to design a broad-spectrum drug for the genus by targeting the *DAHPS* sequence. By analyzing the homology between the protein sequence encoded by *DAHPS* and human protein sequences, *DAHPS*, which does not exist in humans, was proposed to be an important potential antibacterial target. The predicted three-dimensional structure of *Pseudomonas DAHPS* may provide an option for reasonable drug design [48].

Comparative genomics can be used to understand the molecular mechanism of disease and predict targets for new drug design. For example, Zumla et al. [106] discovered that the sequence homology of the severe acute respiratory syndrome coronavirus 2 (SARS-CoV-2) genome with SARS-CoV and Middle East respiratory syndrome coronavirus (MERS-CoV) was about 82%, and the homology of structural proteins was over 90%. The high sequence homology revealed their common pathogenic mechanism. Therefore, the authors of the study designed and developed direct-acting antiviral drugs that target highly conserved enzymes in SARS-CoV-2, such as the main protease (MPRO) or 3C-like protease (3CLpro), the papain-like protease (PLpro), non-structural protein 12 (Nsp12), and RNA-dependent RNA polymerase (RdRP). Among them, ganciclovir and maraviroc, the drugs against MPRO, were considered effective for the treatment of coronavirus disease 2019 (COVID-19) [107].

Comparative genomics is used to find potential therapeutic targets for the development of human drugs and animal drugs. Damte et al. [108] selected five unique pathways of *Mycoplasma hyopneumoniae* strains in KEGG. They then used BLASTp in NCBI to compare the only two protein sequences in the unique pathways with the porcine protein sequences. It was found that the two protein sequences in the unique pathways were not homologous to the porcine protein sequences. Therefore, those essential proteins, which exist in *M. hyopneumoniae* but not in the host (pig), may be useful in drug design and vaccine production against *M. hyopneumoniae*. For more examples of comparative genomics used to identify potential targets, readers can refer to the list of references provided in Table 6.

### Network-Based Methods

Different types of biological networks can be used to predict potential therapeutic targets by network-based methods, such as PPI networks, gene interaction networks and miRNA–mRNA interaction networks. Table 7 lists almost all applications since 2015 of network-based methods to predict potential therapeutic targets, including the databases, software and tools, network types, related pathogens/diseases/processes, and the identified targets. Some of the targets in Table 7 have been verified or used for drug design. Here, we select several examples of previous studies that have used different network types for further description.

**Table 7:** Applications of network-based methods for potential therapeutic target identification

Databases	Software and tools	Network types	Related pathogens/diseases/processes	Predicted targets	References
STRING, MIIP	\	PPIN	*Plasmodium falciparum*	PF10_0232, PFI1475w, PF13_0228	[115]
UniProt, STRING, MINT	Cytoscape	PPIN	Sperm–egg interaction defect	FN1, EGFR, ITGAV, ITGB3, COL1A1, ITGB5	[116]
UniProt, STRING	Cytoscape	PPIN	Cancer	SAGE1, SPO11, MAGEC2, FTHL17, DDX53, BAGE2	[117]
GEO, STITCH, STRING, HPRD, KEGG, SignaLink, OMIM, GAD, FunDO, NHGRI GWAS Catalog	PathwayLinker, Cytoscape, KOBAS	PPIN	Hyperlipidemia	COTL1, VASP, HHAT	[118]
HPRD, GEO, GAD, DO, OMIM, DrugBank, GO, KEGG	\	PPIN	Polycystic ovary syndrome	ESR1, RXRA, NCOA1, NRIP1, ESR2, THRB, RARA, NR0B2, NCOA3, HNF4A, PPARA, PPARG, PPARGC1A, MED1, NR2F1, PNRC2, PGR, ESRRA, ESRRG, RXRB, RARG, VDR	[119]
GO, Swiss-Prot, STRING, BioGRID, DIP, IntAct, BIND, MINT	Cytoscape	PPIN	*Clostridium difficile*	CD2787, CD0237, CD1214, CD2629, CD2643	[120]
STITCH, STRING, GEO	Cytoscape	PPIN	Coronary heart disease	FGG, SLC9A3, MAPK14, FGF1, FGB, F13A1, CASR	[30]
GEO, STRING, GEO, DAVID	Gephi	PPIN	Japanese encephalitis virus	STAT1	[121]
STRING, KEGG, GO	Cytoscape	PPIN	Wound healing	Rela, Nfkb1, Tnfrsf1a	[122]
CellMiner, GEO, HPRD, miRTarBase, TarBase, starBase, SM2miR	CFinder, Cytoscape	PPIN	Ovarian cancer	miR-24-3p, miR-192-5p, miR-139-5p, miR-155-5p	[123]
HPRD, BioGRID, MINT, IntAct, STRING, GEO, CRG	Cytoscape	PPIN	Rheumatoid arthritis	JUN, SYK, LCK	[124]
Google Scholar, PubMed	\	PPIN	Systemic sclerosis	CTGF, HCK, LYN, PDGFRB	[125]
GEO, STRING, JASPAR, TarBase, miRTarBase	Cytoscape, NetworkAnalyst	PPIN	Alzheimer’s disease	PPARG	[126]
DIP, KEGG, DEG, DrugBank	Cytoscape, Gephi	PPIN	*Streptococcus suis*	GlnQ3, GlnQ4, GlnQ1, GlnQ5, PstB1, SSU05_1769	[127]
STRING, UniProt, Pfam	\	PPIN	SARS-CoV-2	MIB1, TBK1, VPS11, AP3B1, GORASP1, GOLGA2	[128]
STRING	Cytoscape	PPIN	Schizophrenia	MAPK1, MAP2K1, CDC42, HSPA1, HSPA8, HRAS, CLTB, SNAP91	[129]
VirusMINT, IntAct, VirusMentha	Cytoscape	PPIN	Influenza A virus	LNX2, MEOX2, TFCP2, PRKRA, DVL2, POLR3F, SNAPC4, GLYR1, ATP6V1G1, PCBP1, EEF1D, DVL3, CREB3	[130]
GEO, STRING	\	PPIN	Pancreatic ductal adenocarcinoma	ITGAV, ITGA2	[131]
TCMSP, TCM, BATMAN-TCM, DRUGBANK, UniProt, GeneCards, OMIM, STRING, KEGG	Cytoscape	PPIN	Colorectal cancer	AKT1, JUN, CDKN1A, BCL2L1, NCOA1	[132]
HuRI, HINT, STRING	Cytoscape	PPIN	Colon adenocarcinoma, glioblastoma multiforme, small cell lung cancer	FANCD2, NCOA4, IKBKB, RHOA	[133]
GEO, miRWalk3, DAVID, STRING	Cytoscape	PPIN	Breast cancer	MAPK1, PRKACA, miR-214-3p, miR-587, miR-4472, miR-4422	[134]
GEO, DAVID, STRING	Cytoscape	PPIN	Steroid-induced osteonecrosis of the femoral head	CXCR1, FPR1, TYROBP, MAPK1	[135]
GEO, DAVID, STRING	Cytoscape	PPIN	Pulmonary arterial hypertension	CCL5, CXCL12, VCAM1, CXCR1, SPP1	[136]
GEO, STRING	Cytoscape	PPIN	Oral squamous cell carcinoma	APP, EHMT1, ACACB, PCNA, PLAU, FST, HMGA2, LAMC2, SPP1	[137]
PATRIC, ARDB, CARD, NDARO, STRING	Cytoscape	GIN	*Pseudomonas aeruginosa* PA01	oprJ, oprM, oprN, ampC, gyrA, mexA, oprD, mexB, nfxB	[31]
NCBI genome, PATRIC, ARDB, CARD, NDARO, STRING, DAVID, TTD	Cytoscape	GIN	*Proteus mirabilis*	rpoB, tufB, rpsl, fusA, rpoC, rpoA	[138]
ARDB, STRING	Cytoscape	GIN	*Enterococcus faecalis* V583	MraY, PbpC, MurE, MurG, MurD	[139]
ARDB, STRING	Cytoscape	GIN	*Salmonella enterica* serovar Typhimurium CT18	tolC, macB, acrA, acrB, mdfA	[140]
STRING	Cytoscape	GIN	*Klebsiella pneumoniae*	gyrA, parC, gyrB, parE, recA	[141]
TCGA, miRCancer, miR2Disease, HMDD, GeneCards, HPAD	\	MMIN	Tumorigenesis	ASPG, AQP2, CNOT8, CTPS1, IFNAR2, MOCS2, PRSS37, VCP	[32]

*PPIN* protein–protein interaction network, *GIN* gene interaction network, *MMIN* miRNA–mRNA interaction network

PPI networks are the most widely used molecular networks in target discovery. For example, Huo et al. predicted proteins FGG, SLC9A3, MAPK14, FGF1, FGB, F13A1, and CASR as potential therapeutic targets for the treatment of coronary heart disease (CHD) by combining the centrality-based and differentia-based approaches [30]. They extracted PPIs related to Danshensu (one of the main active ingredients of *Salvia miltiorrhiza*, known as Danshen) from the STRING database, then integrated the data with the CHD gene expression profile and microarray data obtained from the GEO database to construct a non-CHD state co-expression protein interaction network (CePIN) and a CHD state CePIN on Cytoscape [30]. The non-CHD network contained 91 nodes and 98 edges, and the CHD state CePIN contained 99 nodes and 110 edges [30]. Then, topological analysis and network comparison were performed along with the calculation of network connectivity after the removal of candidate nodes. Finally, two bottleneck proteins, FGG and SLC9A3, existing only in the CHD state CePIN, were selected as the targets of Danshensu in the treatment of CHD and as the potential targets for new drug design [30]. In addition, MAPK14, FGF1, FGB, F13A1, and CASR, obtained through the differentia-based approach, also represented potential therapeutic targets for the treatment of CHD and had been confirmed to be related to CHD to some extent [30].

There are also examples of the use of the centrality based approach alone to identify potential therapeutic targets. For example, Moon et al. generated a list of 1089 differentially expressed genes from patients with diffuse systemic sclerosis by a literature search in Google Scholar and PubMed using specific keywords [125]. Then, using the centrality-based approach to build a PPI network, they identified 1068 interactions of those 1089 genes. Finally, a network centrality analysis identified four hub genes (CTGF, HCK, LYN, PDGFRB) as potential therapeutic targets [125]. In another example, Fathima et al. used non-apoptotic cell death genes of colon adenocarcinoma (COAD), glioblastoma multiforme (GBM), and small cell lung cancer (SCLC) screened from their transcriptome profiles to build three PPI networks [133]. Through centrality analysis, 4 of the top 10 hub proteins, which were not found or only found in one target database, were considered as novel valid therapeutic targets (FANCD2 and NCOA4 for COAD, IKBKB for GBM, and RHOA for GBM and SCLC) [133].

As mentioned above, PPI networks, gene interaction networks, and miRNA–mRNA interaction networks) have applications in predicting potential therapeutic targets. For example, Miryala et al. [31] identified 337 functional interactions of 60 antimicrobial resistance genes of *Pseudomonas aeruginosa* PA01 from the PathoSystems Resource Integration Center (PATRIC) tool, The Antibiotic Resistance Genes Database (ARDB) [142], the comprehensive antibiotic resistance database (CARD), the National database of antibiotic-resistant organisms (NDARO), and the STRING database. By constructing and analyzing the gene interaction network in Cytoscape, nine hub genes were obtained as potential therapeutic targets for new drug development [31]. Xue et al. [32] constructed a miRNA–mRNA interaction network using miRNA and mRNA expression data, and the clinical data of three cancer types downloaded from The cancer genome atlas (TCGA) database [143]. The top 20 miRNAs with the highest degree in each data set were annotated via miRCancer (a microRNA–cancer association database) [144], miR2Disease (a microRNA–disease database) [145], and the Human microRNA Disease Database (HMDD) [146]. After mapping the genes predicted as the targets of more than three miRNAs in the subnetworks to the human protein atlas database (HPAD) [147], eight genes (ASPG, AQP2, CNOT8, CTPS1, IFNAR2, MOCS2, PRSS37, and VCP) were finally identified as potential therapeutic targets [32].

### Comprehensive Applications

In addition to using comparative genomics and network-based methods independently, they can also be combined for target identification. Table 8 lists recent applications of the combined methods for potential therapeutic target identification. We chose three of them as representatives for further description. Nayak et al. screened putative targets for pathogens causing bacterial pneumonia. By bit score, E-value threshold, and sequence length screening of the complete proteome of 13 pathogenic bacterial strains using comparative genomics, 74 proteins non-homologous to human and intestinal flora were identified [103]. An interaction network for the 74 proteins was constructed in Cytoscape, and 12 built-in central parameters of cytoHubba were used to prioritize the nodes, culminating in the identification of 20 genes as hub nodes. Among the 20 genes, 10 have been reported or confirmed as drug targets, and the remaining 10 were considered new potential therapeutic targets for the treatment of bacterial pneumonia [103]. Melak and Gakkhar used BLAST to perform comparative analysis for the H37RV protein-coding genes obtained from the TBDB against DEG and identified 572 essential genes non-homologous with humans [104]. Then, they prioritized the resulting proteins based on centrality measurement in the PPI network, resulting in the identification of 137 central proteins. Combining flux balance analysis of the reactome and structural assessment of targetability, secY (Rv0732), katG (Rv1908c), gltB (Rv3859c), and sirA (Rv2391) were identified as potential therapeutic targets against *Mtb* H37RV [104]. Gupta et al. [148] performed subtractive genomic and comparative genomics of 16 pathogenic *Leptospira* strains retrieved from NCBI against DEG and the Cluster of Essential Genes (CEG) [149] using the Cluster Database at High Identity with Tolerance (CD-Hit) and BLASTp to identify 34 common genes. After analyzing and comparing two extended PPI networks of two strains and multiple sequence alignment, eight proteins (lpxB, lpxK, kdtA, fliN, cobA, metX, thiL, and ubiA) were identified as putative therapeutic targets for drug design or vaccine development [148].

**Table 8:** Applications of comprehensive methods for potential therapeutic target identification

Databases	Software and tools	Related pathogens/diseases	**Predicted targets**	**Ref**
DEG, STRING	BLASTp, Cytoscape	*Listeria monocytogenes* strain EGD-e	dnaN, lmo0162, polC	[150]
TBDB, DEG, STRING	BLASTp	*Mycobacterium tuberculosis* H37Rv	Rv1908c, Rv3795, Rv3793, Rv3794	[104]
TBDB, DEG, STRING	BLAST, Cytoscape	*M. tuberculosis* H37Rv	secY, katG, gltB, sirA	[151]
STRING	Cytoscape	Multi-drug resistant *Clostridium difficile* strain 630	hom, asd, dapG	[152]
DEG, CEG, VFDB, DrugBank, UniProt, DAVID, HPIDB	BLASTp, CD-Hit, BioCyc webserver, Cytoscape	Bacterial pneumonia	manL, cps4L, recU, SP_0645, ezrA, prsA, tarJ, SP_1280, SP_1617, ptsG, dltD, hprK, pepF, coiA, fibB, acpS, manA, mvaK2, mtlD, mtlF	[103]
NCBI, DEG, CEG, UniProt	KAAS, BLASTp, Cytoscape	Leptospira	lpxB, lpxK, kdtA, fliN, cobA, metX, thiL, ubiA	[148]

## Discussion

### Advantages and Prospects of the Two Categories of In silico Methods for Target Identification

Current trends in drug discovery focus on understanding disease mechanisms, followed by target identification and lead compound discovery [5]. Compared with wet experimental methods, in silico methods provide the technology to systematically explore all possible interactions and illuminate the pharmacological patterns [153]. Reliable target identification methods used in conjunction with drug discovery approaches will improve the efficiency of computer-aided drug discovery [5]. Here, we discuss the advantages and prospects of comparative genomics and network-based methods for identifying potential therapeutic targets.

One advantage of comparative genomics is that the definition of essential genes and unique metabolic pathways not only represents the essential issues of biology but is also of great significance in practical applications [111]. Furthermore, with the establishment of Clustered Regularly Interspaced Short Palindromic Repeats (CRISPR) and exome sequencing technology, the number of sequenced human essential genes has increased remarkably [54]. In addition, with the development of bioinformatics and computer science, algorithms have been continuously optimized, generating convenient analysis tools for scientific researchers, and enhancing the potential for comparative genomics in potential therapeutic target identification.

Network-based methods have the advantage of generating visual interactive networks through given databases and are not limited by the lack of quantitative mechanical data [154]. Furthermore, network-based methods do not depend on negative samples and the three-dimensional structure of targets [155], which is time-efficient in the early work of target research. There is also promise that network-based methods will predict more than one target with simultaneous actions, such as a pair of essential proteins [154]. Moreover, network-based methods may be beneficial in identifying candidate multi-target sets in the development of multi-target drugs [156]. Compared with traditional wet experimental methods, which always limit cellular processes to a single component or signaling pathway, network-based methods can be used to identify potential therapeutic targets systematically [15].

### Disadvantages/Limitations of the Two Categories of In silico Methods for Target Identification and Potential Solutions

Although comparative genomics and network-based methods have unique advantages and promising prospects to identify potential therapeutic targets, there are still some drawbacks. For comparative genomics, although this approach is commonly used in the development of drugs against drug-resistant bacteria, the failure rate of old antibiotics is much faster than the development of new antibiotics. Moreover, antibiotics are short-term therapies for the treatment of infections. Additionally, their value is considerably less than the drugs for chronic diseases, so the use of comparative genomics in the development of antibiotics is a long-debated topic [157]. Another issue is that although comparative genomics can reduce the number of experimental targets, making some attractive proteins become potential therapeutic targets, the range of potential targets screened by this method is still very wide and is limited by time and cost. It seems that most of these potential targets screened by comparative genomics will not be used for experimental validation. Therefore, it may be profitable to combine comparative genomics with network-based methods to narrow the scope of experimental targets further and reduce the time and material resources, thereby saving costs in the early stage of drug research and development.

Network-based methods are highly dependent on the accuracy of the source data, potentially requiring a great deal of labor to ensure its accuracy [158]. A promising direction to resolve this problem will be integrating different types and complementary data in the future [6]. Other drawbacks of network-based methods are that they cannot predict proteins or genes without interaction data, and the interactions cannot be quantified [155]. Improved network construction and analysis algorithms or mathematical modeling methods [159] may be required to overcome these issues.

### Comparison and Contrast of the Two Categories of In silico Methods for Target identification

Comparative genomics and network-based methods have unique advantages and disadvantages in predicting targets. Comparative genomics almost exclusively searches within the range of pathogen-associated sequences, limiting the scope to the proteomes closely related to the pathogen. Conversely, network-based methods can be used in pathogens and construct a network for human disease-related proteins or genes. In contrast to comparative genomics, network-based methods can connect long-distance relationships through interactions [160], permitting research into the interplay of evolutionary drivers on a larger scale. Conversely, comparative genomics is usually superior to network-based methods in accuracy because comparative genomics directly compares sequences, which are always constant and almost have no deviation. However, there may be false positives and false negatives in the interaction data used in network-based methods [161], and the interactions are only qualitative [160], which may lead to bias. In summary, the combined use of comparative genomics and network-based methods may be more beneficial than either method alone to improve the accuracy and efficiency in target identification.

### Previous Reviews and Prospective Studies on In silico Methods for Target Identification

We have collected five reviews on in silico methods for identifying potential therapeutic targets during 2016–2020, which will be briefly discussed in this section. Sekyere and Asante [7] reviewed comparative genomic analysis *trans*-complementation assays in the context of antibiotic resistance research and new drug discovery by describing the emergence of several new drug resistance genes, such as *lsa*(C), *erm*(44), *VCC-1*, *mcr-1*, *mcr-2*, *mcr-3*, *mcr-4*, *bla*_*KLUC-3*_, and *bla*_*KLUC-4*_. For readers interested in further understanding pathogen protein targets, Saha et al. reviewed the computational work and functional prediction from PPI networks applied to different infectious diseases with *Plasmodium falciparum* used as an example to analyze the process of protein target identification through the host–pathogen protein interactions [162]. Katsila et al. [5] surveyed chemical informatics and network-based methods for identifying therapeutic targets and introduced some databases and network computing tools for target identification. They also appraised the process of computer-aided drug design (CADD), including ligand-based drug design and structure-based drug design [5]. Readers interested in CADD can peruse their article for further understanding. Reisdorf et al. introduced database resources for identification, prioritization, and validation of disease targets, including emerging integrated bioinformatics platforms, such as Open Targets, and public resources, such as DrugBank and ChEMBL [163]. In comparison, the database resources we described focus more on classic or commonly used databases for applications. We also recommend the review by Agamah et al. [153], which examined current in silico methods for the identification of therapeutic targets and candidate drugs, including network-based analysis approaches, data mining, reverse docking, biospectra analysis, and ligand-based in silico target prediction and compared the different approaches and propounded the benefits of hybrid approaches.

### Related Methods for Target Identification

In silico subtractive genomics (first mentioned in Sect. 2.1), also known as differential proteome mining, is a comparative genomics-based method [164]. Subtractive genomics gradually subtracts proteins from the complete proteome of pathogens to find rational targets [18]. The difference between subtractive genomics and comparative genomics is in the range of application of the two methods. Subtractive genomics has been widely used for developing potential anti-pathogen infection drugs [18], whereas comparative genomics can be used not only to identify potential targets of pathogens but also to understand the molecular basis of disease [106].

For network-based methods, in addition to the centrality-based and differentia-based approaches we reviewed above, there are also studies showing the use of network influence [165], controllability [166], and topological similarity strategy [167] in target identification, but the relevant applications are much fewer. Compared with network centrality, the network influence strategy focuses on the vulnerable nodes close to the central nodes in networks. Acting on these nodes may not be fatal but can have a major impact on the central nodes, so these nodes have the potential to be therapeutic targets [165]. The controllability strategy applies structural controllability theory to determine the minimum set of driver nodes in control of the entire network and identify indispensable nodes as prime targets for disease-causing mutations, viruses, and drugs [166]. The topological similarity strategy focuses on the nodes in the network with similar topological properties to the existing drug targets, which can be potentially developed as therapeutic targets [167].

Commonly used experimental methods for potential therapeutic target identification, especially for essential genes, include single-gene knockout, antisense RNA inhibition of gene expression, large-scale transposon mutagenesis, and CRISPR/Cas9 nuclease system knockout screening.The limitations of experimental methods in identifying essential genes are listed in Table 9 [168].

**Table 9:** Limitations of experimental methods in identifying essential genes

Experimental methods	Limitations
Single-gene knockout strategy	Requires detailed genome annotation
Antisense RNA inhibition method	Requires detailed genome annotation
Transposon mutagenesis	Missing low-abundance transcripts, low resolution in locating insertion sites, and narrow ranges in counting probe density

Current computational studies are based on the integration of prior knowledge, the sparseness of which is still limiting the integrality and accuracy of computational prediction [169]. Data reproducibility of in silico methods is also an essential issue but might be improved by external validation and detailed reports of experimental datasets [153]. It should be emphasized that computational methods complement laboratory-based methods and that the targets identified by in silico methods need to be experimentally validated.

### Review and Prospection of Deep Learning Architecture in Target Identification

Deep learning (DL), a relatively new computational technique that has become a hot research topic, has been rapidly developed and widely used to predict potential therapeutic targets. DL is a subclass of machine learning (ML) algorithms. It uses artificial neural networks with many layers of nonlinear processing units for learning data representations [170]. Therapeutic target identification based on ML or DL is usually used to predict targets of drug repositioning, which means to predict new targets for existing drugs. There are two steps in the ML method to predict therapeutic targets. First, the compounds are transformed into an effective representation, a process called input features, followed by the construction of the feature vectors as input for the ML algorithm to learn the functional relationship between the input feature and the target property [171]. Compared with ML methods, DL reconstructs the original input information into a distributed representation through neurons in the hidden layer. Another characteristic of DL models is that they can automatically learn features upon completing classification and other tasks and learn more complex features when the number of layers increases. DL architectures are well-suited for target prediction because they allow for multitask learning and automatically construct complex features, which, for target prediction, are assumed to be pharmacophore descriptors. Multitask learning has the advantage of allowing for multi-label information and can, therefore, utilize relations between targets. It also permits hidden unit representations to be shared among prediction tasks, which is particularly valuable because some targets have very few measurements available, making single-target prediction ineffective. In addition, DL can boost the performance of tasks with a few training examples. The other advantage of deep networks is that they provide hierarchical representations of a compound, where higher levels represent more complex properties [172].

Convolutional neural networks (CNNs) are a representative DL architecture in potential target prediction. CNNs contain convolutional layers, pooling layers, and fully connected layers. Convolutional layers and pooling layers are responsible for the feature extraction, and fully connected layers are used to construct the nonlinear relationship of the extracted features for obtaining the output [171]. Another DL architecture is deep neural networks (DNNs), which contain multiple hidden layers, with each layer comprising hundreds of nonlinear process units. DNNs can deal with many input features, and the neurons in different layers of a DNN can automatically extract features at different hierarchical levels [173]. The third main DL architecture is auto-encoders, which is a neural network used for unsupervised learning. Auto-encoders contain an encoder part that transforms the input information into a limited number of hidden units and then couples a decoder neural network with the output layer having the same number of nodes as the input layer [174].

Several studies have reported DL for therapeutic target prediction in recent years [175–177]. For example, Wang et al. [178] constructed a framework that combines a biased support vector machine and a stacked auto-encoder DL model to identify drug target proteins. The stacked auto-encoders were trained to extract properties from the original protein representations, and the biased support vector machine was used to perform the potential target identification task. The framework identified 23% of the original non-drug target proteins as possible therapeutic target proteins. Zeng et al. [179] developed a DL method, named deepDTnet, for novel target identification. A DNN algorithm was used to learn the relationships between drugs and targets. The model was used to predict the new target for topotecan (an approved topoisomerase inhibitor of human retinoic-acid-receptor-related orphan receptor-gamma t, ROR-γt). Human ROR-γt was predicted as the target, and bioassay experiments showed high inhibitory activity (IC_50_ = 0.43 μM) on ROR-γt. Lee et al. [180] proposed a DL model named DeepConv-DTI (deep learning with convolution on protein sequences for prediction of drug–target interaction) based on CNN for drug–target interactions prediction, which can be used for target identification. The training dataset contained 11,950 compounds, 3,675 proteins, and 32,568 drug–target interactions. The CNN model is constructed to capture local residue patterns and concatenate protein features with drug features through the fully connected layers. The hyperparameters with an external validation dataset were then optimized. The possible drug–protein interactions are output.

Although DL has advantages in recognition, classification, and feature extraction from complex and noisy data, it still has limitations. First of all, DL is a “black box,” which makes it hard to explain the prediction result and inherent principles of why the compound is effectively targeted to the predicted target. Second, it needs a large number of experimental datasets of drug–target relationships for its training. However, there is currently a lack of experimental data of drug–target relationships [181]. Consequently, there is a risk of overfitting when training the model, leading to low accuracy of the prediction result. Third, DL is usually computationally intensive, time-consuming, and often requires access to and programming knowledge for graphics processing units. DL has recently been applied successfully in therapeutic target identification. However, due to the lack of large-scale studies or experimental data and the hyperparameter selection bias that comes with the high number of potential DL architectures, DL still has scope for improvement and development in research to predict potential therapeutic targets [172, 182].

## Conclusion

In this review, we introduced, in detail, the two categories of in silico methods for potential therapeutic target identification—comparative genomics and network-based methods—and summarized the databases and software commonly used for these approaches. We also collected and highlighted some previous applications of these methods for therapeutic target identification. Additionally, we analyzed the advantages and disadvantages of the methods and their application prospects. Finally, we accentuated the characteristics of our review in the context of previously published relevant reviews and methods. The purpose of this review was to help readers quickly understand the rationales of in silico methods for potential therapeutic target identification, and become familiar with the available tool resources and the applications of these methods, to harness the full use of the existing tools for target prediction. We strongly believe that more accurate predictions due to users’ familiarity with existing resources will increase the importance of computational methods in the identification of potential therapeutic targets for future research. In turn, the failure rate due to target problems in drug development, the input–output ratio of drug discovery, and the cost of subsequent experiments can be expected to reduce and the drug development cycle time to shorten.
